# Orientable Surfactants on Thin Liquid Films: A Dynamic Density-Functional Theory Approach

**DOI:** 10.1021/acs.jpcb.6c00971

**Published:** 2026-09-11

**Authors:** Toby Kay, Serafim Kalliadasis

**Affiliations:** Complex Multiscale Systems Group, Department of Chemical Engineering, 4615 Imperial College, London SW7 2AZ, U.K.

## Abstract

Thin liquid films are ubiquitous across many natural and engineering systems, including films which are laden with surface-active molecules, i.e., surfactants. The presence of surfactants may have a destabilizing effect on the film owing to their influence on surface tension, the so-called Marangoni effect, which in turn can induce flows in the film. Classical thin-film models for surfactant-laden films lead to paradigmatic gradient-dynamics equations governing the film height and surfactant concentration and have been widely studied. However, in all these works, which are based on fluid dynamics or nonequilibrium thermodynamics, the shape of surfactants is neglected, and they have been treated as symmetric point-like particles. In general, this is a drastic oversimplification, as surfactants are amphiphilic with a polar head–tail structure. To account for this effect we use elements from the statistical mechanics of classical fluids, namely, density-functional theory (DFT), and its dynamic extension (DDFT). Starting from DDFT and under the long-wave approximation, we derive the pertinent thin-film equations with the surfactants treated as polar uniaxial particles. These are equations which govern the film height, as well as the surfactant concentration and polarization field. They preserve the gradient-dynamics form by appropriately defining the free energy, which contains the usual interfacial contributions, as well as further contributions from the polarization field. In doing so, we uncover a novel form of a generalized surface tension that is dependent on the surfactant polarization, as well as concentration, and show that it arises in a thermodynamically consistent way.

## Graphical abstract

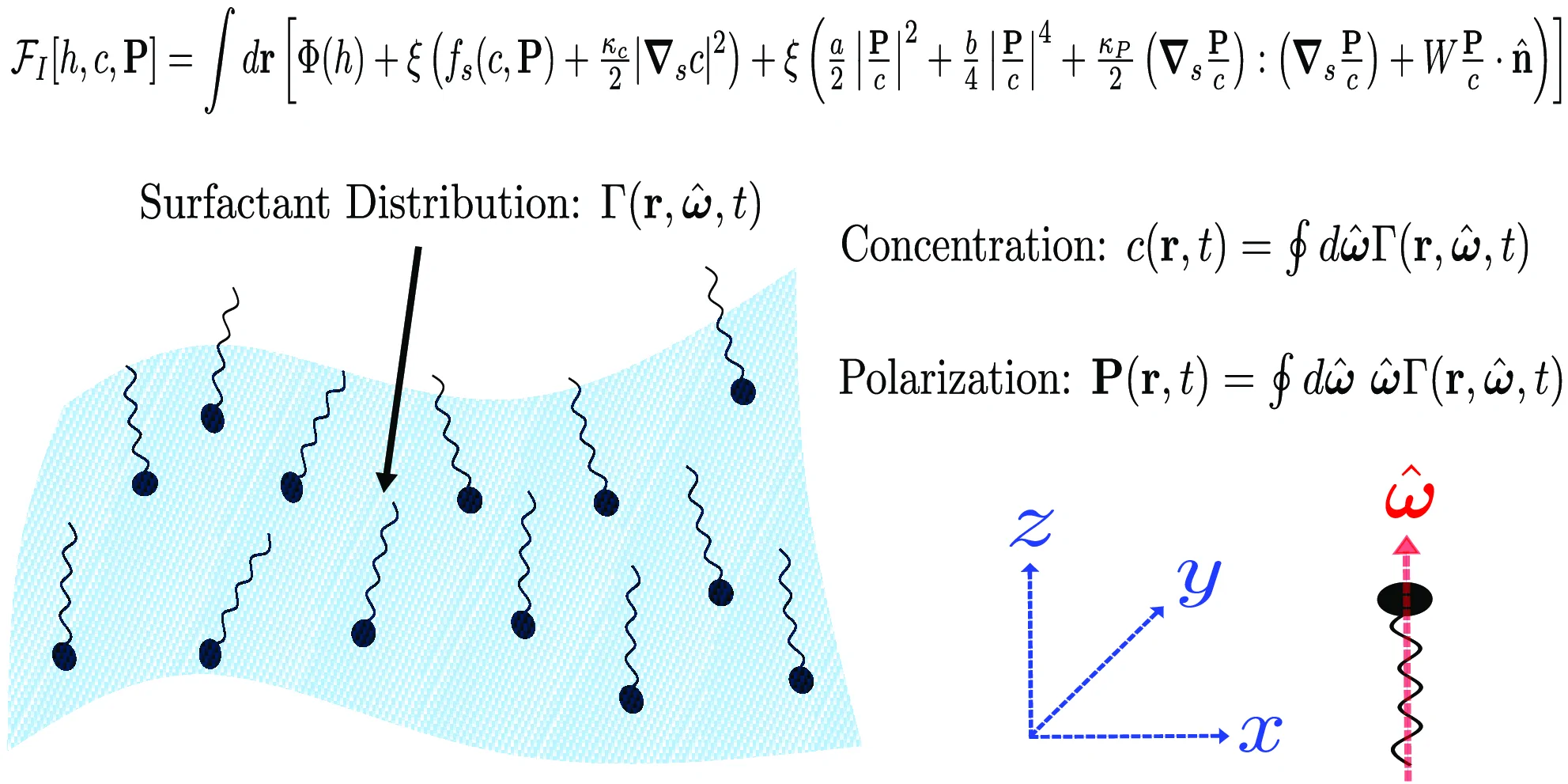

## Introduction

Thin-liquid films with surface active agents, so-called surfactant-laden films, are prevalent in technology and nature; from industrial and commercial applications with foams, emulsions^1^ and dip coatings,^2^ to biological and physiological settings such as tear films in the eye^3^ and surfactant layers in the alveoli of the lung.^4^ Surfactants are known to profoundly affect interfacial flows. The so-called solutocapillary Marangoni effect, is the appearance of flow, or the altering of an existing flow, due to surface tension nonuniformity, i.e., when the surface tension varies along an interface with surfactant concentration.^5,6^ This effect can be seen in the well-known phenomena of the “tears of wine”.^7^ Not surprisingly, surfactant-laden films have received considerable attention over the years.

Typically, their dynamics is governed by two coupled evolution equations for the height of the film and the surfactant concentration. Two main approaches to deriving these have been utilized. The first comes from fluid dynamics, where one starts with the Navier–Stokes equations of motion and associated wall and free-surface boundary conditions, together with a surface advection-diffusion equation for the surfactant concentration,^8^ generalized in refs 9,10 . One then applies the long-wave or lubrication approximation^11^ to obtain a set of two coupled evolution equations for the film height and surfactant, e.g., refs 12–14 . Alternatively, one may follow a nonequilibrium thermodynamic approach and construct the set of coupled evolution equations in terms of an interfacial free-energy functional, highlighting the gradient dynamics form of the equations, and a mobility matrix^15–17^ (in the same spirit with diffuse-interface methods). With a suitable choice for the free-energy functional and mobility matrix both approaches lead to equivalent sets of equations.

Surface-active agents are amphiphilic molecules with a head–tail structure, where the head and tail are typically hydrophilic and hydrophobic, respectively, enabling interaction with both polar and nonpolar solvents. This structure gives them a tendency to orient in specific ways on the surface of a liquid film. In previous works the shape of the surfactant molecule has been neglected, and surfactant molecules are instead treated as symmetric point-like particles. This may be a valid approximation in the dilute regime, where surfactant molecules may be treated as an ideal gas. However, this is no longer valid for higher surfactant concentrations as the interactions between the surfactant molecules and the film itself become increasingly important and surfactant orientation has a significant effect on these interactions. In the case of a flat-interface two-phase fluid system with soluble surfactants, it has recently been shown^18,19^ that the orientation of the surfactant has a significant effect on the interfacial dynamics, specifically the orientation induced altering of the effective interface tension. This implies that for free-surface films the orientation of the surfactants will have an impact on the Marangoni stress and, consequently, on the dynamics of the film height and surfactant concentration.

In the present study we explicitly account for the shape of surfactants, specifically their uniaxial polar structure, a significant departure from previous works. To do so we turn to statistical mechanics of classical fluids, namely density functional theory (DFT) and its dynamic extension (DDFT). We start by introducing the DDFT for uniaxial colloidal particles in a fluid and extend this to when the particles are confined to the surface of a film, thus obtaining a microscopic derivation of the pertinent surfactant transport equation. Building on previous surfactant-laden thin-film derivations, we apply the long-wave approximation and obtain a coupled pair of evolution equations governing the height of the thin film and the joint surfactant position and orientation density. Defining the surfactant concentration and polarization as moments of this density, we find the governing equations of these quantities which are coupled to the film-height equation. To close this set of equations, we define the free-energy functional in terms of the usual interfacial contributions as well as extra terms arising from the polarization of the surfactants. Finally, by defining a generalized surface tension in terms of the local grand-potential density, we are able to write the set of thin-film equations in a gradient dynamics form in terms of the free-energy functional.

## Surface DDFT for Uniaxial Particles

Figure 1 depicts the physical setting. A coordinate system (*x*, *y*, *z*) is chosen so that *x* and *y* are the streamwise and spanwise coordinates, respectively, and *z* is the outward-pointing coordinate normal to the wall. The wall is then located at *z* = 0 and the film free surface at *z* = *h*(**r**, *t*), where **r** = (*x  y*)^T^. The film is at a constant temperature *T*, and its free surface is laden with surfactant molecules. These surface-active agents are supposed to be of a generic amphiphilic head–tail structure, such that they may be treated as being polar uniaxial particles. We will also assume that they are insoluble but they diffuse on the film surface and also assume that their surface concentration is high enough that molecular interactions between the surfactant molecules cannot be neglected.

**Figure 1:**
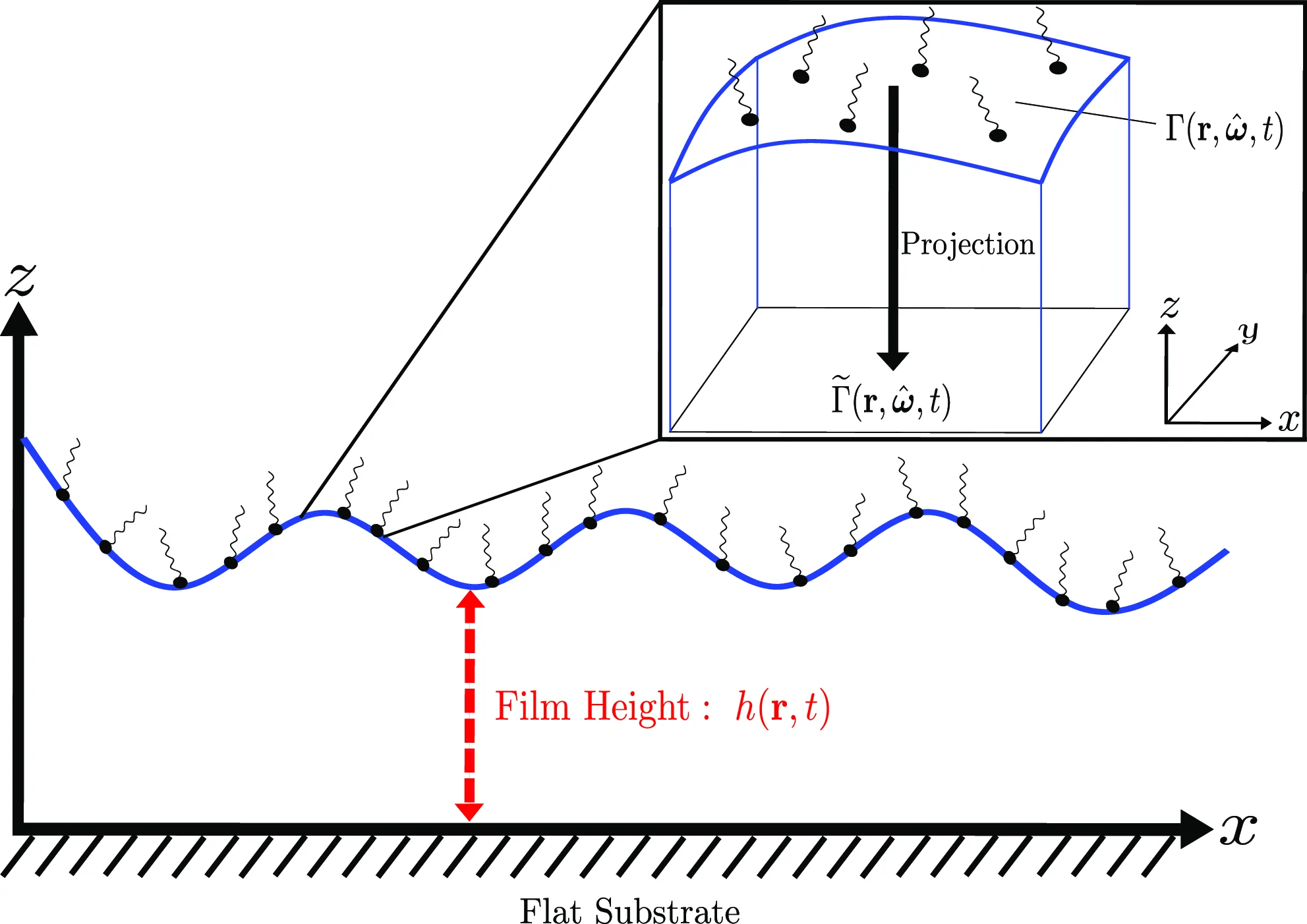
Sketch of the profile geometry for a thin liquid film on a planar horizontal substrate.

First, we introduce the DDFT for polar uniaxial colloidal particles that undergo Brownian motion in a generic three-dimensional, *d* = 3, fluid^20,21^

∂tρ(r,ω̂,t)=∇r·D(ω̂)·[ρ(r,ω̂,t)∇rδF[ρ]δρ(r,ω̂,t)]+Drotω̂×∇ω̂·[ρ(r,ω̂,t)ω̂×∇ω̂δF[ρ]δρ(r,ω̂,t)] 1

where $r={\left(x\;y\;z\right)}^{T}$ is the *d* = 3 positional coordinate and **ω̂** is a unit vector giving the particle orientation pointing from tail to head, such that in the case of apolar particles, **ω̂** ≡ −**ω̂**. $\nabla_{r}$ is the Cartesian gradient operator and ***∇***_**ω̂**_ is the gradient on the unit sphere, such that **ω̂** × **∇**_**ω̂**_ is the rotation operator.^20^ $D_{\mathrm{rot}}$ is the rotational diffusion coefficient and the translational diffusion tensor for uniaxial particles is given by^20^

2 $$D\left(\mathrm{\omega ̂}\right)=D_{∥}\mathrm{\omega ̂}⊗\mathrm{\omega ̂}+D_{⊥}\left(I-\mathrm{\omega ̂}⊗\mathrm{\omega ̂}\right)$$

where ⊗ is the dyadic product, and *D*_∥_ and *D*_⊥_ are the diffusion coefficients parallel and perpendicular to the axis of the particle, respectively, and **I** is the identity matrix. ρ(r,ω̂,t) describes the one-body position and orientation distribution of the colloidal particles (corresponding to density in fluid dynamics). F[ρ] is the free-energy functional, which for now we take it to be just a functional of the distribution ρ(r,ω̂,t) , which is of the standard form,^22–24^ $F=F_{\mathrm{id}}+F_{\mathrm{exc}}+F_{\mathrm{ext}}$ , where we have contributions from the ideal gas, excess and external free energies, respectively. At equilibrium the ideal and external free-energy functionals are given, respectively, by^20^

$$F_{\mathrm{id}}\left[\rho_{\mathrm{eq}}\right]=k_{B}T\int dr\oint d\mathrm{\omega ̂}\rho_{\mathrm{eq}}\left(r,\mathrm{\omega ̂}\right)\left[\ln V\rho_{\mathrm{eq}}\left(r,\mathrm{\omega ̂}\right)-1\right]$$ 3a

$$F_{\mathrm{ext}}\left[\rho_{\mathrm{eq}}\right]=\int dr\oint d\mathrm{\omega ̂}\rho_{\mathrm{eq}}\left(r,\mathrm{\omega ̂}\right)V_{\mathrm{ext}}\left(r,\mathrm{\omega ̂}\right)$$ 3b

where V is the thermal volume of the particle, *V*_ext_ is the external potential, and ∮*d* **ω̂** is the integral over the unit sphere. For the excess free energy we have the relations^20^

4a $$\rho_{\mathrm{eq}}\left(r,\mathrm{\omega ̂}\right)\nabla_{r}\frac{\delta F_{\mathrm{exc}}\left[\rho_{\mathrm{eq}}\right]}{\delta \rho_{\mathrm{eq}}\left(r,\mathrm{\omega ̂}\right)}=\int dr^{\prime }\oint d{\mathrm{\omega ̂}}^{\prime }\rho_{\mathrm{eq}}^{\left(2\right)}\left(r,r^{\prime },\mathrm{\omega ̂},{\mathrm{\omega ̂}}^{\prime }\right)\nabla_{r}U\left(r,r^{\prime },\mathrm{\omega ̂},{\mathrm{\omega ̂}}^{\prime }\right)$$

4b $$\rho_{\mathrm{eq}}\left(r,\mathrm{\omega ̂}\right)\mathrm{\omega ̂}\times \nabla_{\mathrm{\omega ̂}}\frac{\delta F_{\mathrm{exc}}\left[\rho_{\mathrm{eq}}\right]}{\delta \rho_{\mathrm{eq}}\left(r,\mathrm{\omega ̂}\right)}=\int dr^{\prime }\oint d{\mathrm{\omega ̂}}^{\prime }\rho_{\mathrm{eq}}^{\left(2\right)}\left(r,r^{\prime },\mathrm{\omega ̂},{\mathrm{\omega ̂}}^{\prime }\right)\mathrm{\omega ̂}\times \nabla_{\mathrm{\omega ̂}}U\left(r,r^{\prime },\mathrm{\omega ̂},{\mathrm{\omega ̂}}^{\prime }\right)$$

where $\rho_{\mathrm{eq}}^{\left(2\right)}\left(r,r^{\prime },\mathrm{\omega ̂},{\mathrm{\omega ̂}}^{\prime }\right)$ is the joint equilibrium density and $U\left(r,r^{\prime },\mathrm{\omega ̂},{\mathrm{\omega ̂}}^{\prime }\right)$ is the interaction potential. Although we are interested in out-of-equilibrium systems we make the adiabatic approximation,^23–26^ by which we approximate the higher-body correlations by those of an equilibrium fluid with the same density; this is effectively an equilibrium sum rule, by which we assume the system is sufficiently close to equilibrium and evolves slowly enough that the ideal gas and external free-energy functionals in eqs 3 and 4 and functional derivatives of the excess free-energy functional in eqs 5 and 6, may be adequately used in the time-dependent system of interest. It should be noted though, that, in general, the adiabatic approximation is uncontrolled and that superadiabatic effects are generally present in out-of-equilibrium systems as detailed in refs 27,28 .

As our focus is the motion of surfactants on the surface of the film we need to adapt eq 1 to account for lower dimensionality and the influence of the film dynamics. Related approaches include (lower-dimensional) DDFT applied to confined geometries,^29–32^ as well as studies on transport of anisotropic colloidal particles at interfaces.^33–36^ In our case, we consider the density to be describing the motion of polar uniaxial colloidal particles constrained to the film surface, defined by the film height *z* = *h*(**r**, *t*). It is usual to define the joint surfactant concentration and orientation density as the dimensionless quantity, $\Gamma \left(r,\mathrm{\omega ̂},t\right)=l^{2}\rho \left(r,\mathrm{\omega ̂},t\right)$ , where $l^{2}$ is the molecular length scale, i.e., the surface area per surfactant molecule when there is maximum packing on the surface.^15^ From eq 1 and ref 8 we are able to formulate the following transport equation governing the surfactant concentration on the free surface, (details are given in Appendix A)­

5 $$\partial_{t}\Gamma =-\nabla_{s}\cdot \left(\Gamma v_{s}\right)-\Gamma \left(\nabla_{s}\cdot \mathrm{n̂}\right)\left(v\cdot \mathrm{n̂}\right)+\nabla_{s}\cdot D_{s}\cdot \left[\Gamma \nabla_{s}\frac{\delta F}{\delta \Gamma }\right]+D_{\mathrm{rot}}\mathrm{\omega ̂}\times \nabla_{\mathrm{\omega ̂}}\cdot \left[\Gamma \mathrm{\omega ̂}\times \nabla_{\mathrm{\omega ̂}}\frac{\delta F}{\delta \Gamma }\right]$$

where we have implicitly included the rescaling by $l^{2}$ . Here **v** represents the interfacial film velocity and **v**_*s*_ = (**I** – **n̂** ⊗ **n̂**) · **v** is the velocity tangential to the film surface, where

$$\mathrm{n̂}=\frac{{\left(-\partial_{x}h\;-\partial_{y}h\;1\right)}^{T}}{\sqrt{1+|\nabla h{|}^{2}}}$$ 6

is the unit normal (outward-pointing) to the surface. Then $\nabla_{s}=\left(I-\mathrm{n̂}⊗\mathrm{n̂}\right)\cdot \nabla_{r}$ is the surface gradient and **D**_*s*_ = (**I** – **n̂** ⊗ **n̂**) · **D** · (**I** – **n̂** ⊗ **n̂**) is the translational diffusion tensor projected onto the surface. In eq 7 we leave the rotation term unmodified as compared to eq 1, as we allow the surfactant molecules to rotate freely in three dimensions.

There is a rather subtle point associated with the surfactant transport equation in [Disp-formula eq7], and more general, a transport equation for an interfacial quantity, such as mass, mole, or charge density, Γ. The partial time derivative, ∂Γ/∂*t*, has to be defined with care. Specifically, in ref 10, a generalized transport equation for an interfacial entity was derived. In this equation, ∂Γ/∂*t* is interpreted as the partial time derivative of Γ with respect to a local frame of reference moving with the velocity normal to the surface, **v**_n_: it is the “normal time derivative”, (∂Γ/∂*t*)_n_. This transport equation agrees with the one derived in ref 8 provided that the time derivative in ref 8 is not interpreted as the usual partial time derivative, i.e., the derivative obtained by differentiating with respect to time for a fixed point in space, hence the time derivative in the laboratory frame, but as (∂Γ/∂*t*)_n_. The same conclusion was reached in ref 9 but with a more involved derivation to that in ref 10.

## Long-Wave Expansion

Equation 7 is quite intricate, but it can be greatly simplified in the framework of the long-wave approximation.^11^ The approximation is widely known and has been used successfully utilized for a wide range of thin-flow settings in the presence of various levels of complexity, including thermo-/solutocapillary Marangoni effects and substrate topography, e.g., refs 12,37 . It relies on the assumption that waves on the surface of the film, are typically long compared to the film thickness, or equivalently, deformations of the free surface are weak. They are also slow in time. Equivalently, the evolution of thin films is dominated by long-wave modes.^11^ This is because the viscosity of the fluid ensures a strong coherence of the flow across the film. As a consequence, variations of the interface are slow in time and space.

These fortuitous characteristics, inherent to thin films, enable a drastic reduction of the complexity of the governing equations and boundary conditions, and to obtain systems of simplified model equations. To express the smallness of the interfacial slope, a small parameter *ε* ∼ |**∇** *h*| ≪ 1 is introduced, such that ∂_*x*,*y*,*t*_ ∼ *ε*. The film can then be seen as sufficiently flat, i.e., **n̂** ≈ (0 0 1)^T^ to leading order in *ε*, and evolving sufficiently slowly. Under this approximation, we have that the interfacial velocity **v**, is dominated by its tangential components. Therefore, the normal component is small in comparison, which leads to the term Γ­(**∇**_*s*_ · **n̂**)­(**v** · **n̂**) in eq 7 only contributing at higher orders and can thus be neglected. Similarly, at leading order the surface gradient can be replaced by the *d* = 2 Cartesian gradient, **∇**_**r**_, leading to the long-waveform of eq 7

7 $$\partial_{t}\Gamma =-\nabla_{r}\cdot \left(\Gamma v_{s}^{\mathrm{xy}}\right)+\nabla_{r}\cdot D^{\mathrm{xy}}\cdot \left[\Gamma \nabla_{r}\frac{\delta F_{I}}{\delta \Gamma }\right]+D_{\mathrm{rot}}\mathrm{\omega ̂}\times \nabla_{\mathrm{\omega ̂}}\cdot \left[\Gamma \mathrm{\omega ̂}\times \nabla_{\mathrm{\omega ̂}}\frac{\delta F_{I}}{\delta \Gamma }\right]$$

where **v** ^*xy*^ = (*v*_*x*_ *v*_*y*_)^T^ is the film velocity vector parallel to the substrate and **v**_*s*_^*xy*^ = **v** ^*xy*^(*h*) is the corresponding velocity at the interface. **D** ^*xy*^ is the (2 × 2) diffusion tensor with only the *x*- and *y*-components retained. $F_{I}$ is the interfacial free energy which will have additional contributions due to surface dynamics as compared to the general free energy, F , introduced in the previous section.

Now, we calculate the velocity, **v** ^*xy*^ that appears in eq 9. This is a standard calculation using the long-wave expansion, where one can show that **v** ^*xy*^ satisfies the Stokes equation, e.g., refs 11, 12, and 16

8 $$\eta \partial_{z}^{2}v^{\mathrm{xy}}\left(z\right)=\nabla_{r}p$$

where η is the (bulk) viscosity and *p* is the pressure. Equation 10 may be trivially solved by a double integration over *z*, and employing the no-slip boundary condition on the substrate, **v** ^*xy*^(0) = 0, as well as the leading-order stress balance condition on the interface, η∂_*z*_ **v** ^*xy*^(*h*) = ***∇***_**r**_γ, where γ is the interfacial tension. One then obtains the following expression for the velocity,

9 $$v^{\mathrm{xy}}\left(z\right)=-\frac{z}{\eta }\left[\left(h-\frac{z}{2}\right)\nabla_{r}p-\nabla_{r}\gamma \right]$$

such that the surface velocity is given by,

10 $$v_{s}^{\mathrm{xy}}=-\frac{h^{2}}{2\eta }\nabla_{r}p+\frac{h}{\eta }\nabla_{r}\gamma$$

From eq 11 we are able to write the governing equation for the height of the film, *h*(**r**, *t*), via the conservation law or continuity equation,

$$\partial_{t}h=-\nabla_{r}\cdot \int_{0}^{h}dzv^{\mathrm{xy}}\left(z\right)$$ 11

which gives the well-known form, e.g., refs 2,13,15–17 .

12 $$\partial_{t}h=\nabla_{r}\cdot \left[\frac{h^{3}}{3\eta }\nabla_{r}p-\frac{h^{2}}{2\eta }\nabla_{r}\gamma \right]$$

We may relate the pressure in eq 14 to the free-energy functional by explicitly including the dependence of the free energy on the height, and concentration Γ, as $F_{I}\left[h,\Gamma \right]$ , such that^16^

13 $$p\left(r,t\right)=\frac{\delta F_{I}\left[h,\Gamma \right]}{\delta h\left(r,t\right)}$$

However, the consequence of this is that the height *h* and concentration Γ are not independent as Γ is the concentration on the curved surface, defined by *h*.^2^ To remedy this, we take Γ̃ = *ξΓ*, where $\xi =\sqrt{1+|\nabla_{r}h{|}^{2}}$ is the metric factor of the film surface, such that Γ̃ is the surfactant concentration projected onto the flat substrate plane, as illustrated in Figure 1. Therefore, we write the free-energy functional as $F_{I}\left[h,\mathrm{\Gamma ̃}/\xi \right]$ , and take the variations with respect to Γ̃. Similarly, we may use eq 12 in eq 9 to write the governing equation for Γ­(**r**, **ω̂**, *t*),

14 $$\partial_{t}\Gamma =\nabla_{r}\cdot \left[\frac{h^{2}\Gamma }{2\eta }\nabla_{r}\frac{\delta F_{I}}{\delta h}-\frac{h\Gamma }{\eta }\nabla_{r}\gamma \right]+\nabla_{r}\cdot D^{\mathrm{xy}}\cdot \left[\Gamma \nabla_{r}\frac{\delta F_{I}}{\delta \mathrm{\Gamma ̃}}\right]+D_{\mathrm{rot}}\mathrm{\omega ̂}\times \nabla_{\mathrm{\omega ̂}}\cdot \left[\Gamma \mathrm{\omega ̂}\times \nabla_{\mathrm{\omega ̂}}\frac{\delta F_{I}}{\delta \mathrm{\Gamma ̃}}\right]$$

which is coupled to the governing equation for the film height, eq 14.

To further simplify eq 16, we must obtain the form of the functional $F_{I}$ which will include interfacial contributions as well contributions from the dynamics of the surfactants, as in eqs (3) and (4). Due to the inclusion of the surfactant orientation in Γ, we may also add in the free-energy functional orientation-derived effects on the interface. To do so, we coarse-grain by considering the first two moments of Γ with respect to **ω̂**. First we have the zeroth-moment, the marginal of Γ over **ω̂**, as the surface concentration *c*(**r**, *t*) where

15 c(r,t)=∮dω̂Γ(r,ω̂,t)

and the first-moment as the expectation over **ω̂** as,

16 P(r,t)=∮dω̂ω̂Γ(r,ω̂,t)

which defines the polarization vector field of the surfactants. The polarization gives the local average orientation of the surfactant molecules, such that it measures how strongly and in which direction the surfactant molecules are pointing. Clearly, for apolar molecules **P** will vanish, but surfactant molecules, with their head–tail structure, are polar, and **P**, in general will be nonzero. If we make the assumption that the orientation-based contributions are wholly defined by the polarization, **P**, we are able to approximate the free-energy functional as

17 $$F_{I}\left[h,\Gamma \right]\approx F_{I}\left[h,c,P\right]$$

By using eq 19 in eq 16 and performing the integrations in eqs 17 and 18 we are able to derive thin-film equations for the surfactant concentration and polarization.

## Thin-Film Equations for Surfactant Concentration and Polarization

To perform the coarse-graining of eq 16 to find the governing equations of *c* and **P**, we must first express the variational derivatives of the free energy functional in terms of c̃ = *ξc* and **P̃** = ξ**P**. To do so, we use the fact that c̃ and **P̃** are functionals of  Γ̃, which means we may calculate the variational derivatives using the functional chain rule, such that

18 δFIδΓ̃(r,ω̂,t)=∫dr′[δFIδc̃(r′,t)δc̃(r′,t)δΓ̃(r,ω̂,t)+δFIδP̃(r′,t)·δP̃(r′,t)δΓ̃(r,ω̂,t)]

From eqs 17 and 18, we have that

19a $$\frac{\delta \mathrm{c̃}\left(r^{\prime },t\right)}{\delta \mathrm{\Gamma ̃}\left(r,\mathrm{\omega ̂},t\right)}=\oint d{\mathrm{\omega ̂}}^{\prime }\delta \left(r-r^{\prime }\right)\delta_{\mathrm{rot}}\left(\mathrm{\omega ̂},{\mathrm{\omega ̂}}^{\prime }\right)=\delta \left(r-r^{\prime }\right)$$

19b $$\frac{\delta \mathrm{P̃}\left(r^{\prime },t\right)}{\delta \mathrm{\Gamma ̃}\left(r,\mathrm{\omega ̂},t\right)}=\oint d{\mathrm{\omega ̂}}^{\prime }{\mathrm{\omega ̂}}^{\prime }\delta \left(r-r^{\prime }\right)\delta_{\mathrm{rot}}\left(\mathrm{\omega ̂},{\mathrm{\omega ̂}}^{\prime }\right)=\mathrm{\omega ̂}\delta \left(r-r^{\prime }\right)$$

where δ_rot_(**ω̂**, **ω̂**′) is the rotational Dirac-δ such that for an arbitrary function *g*(**ω̂**), ∮*d* **ω̂**′*g*(**ω̂**′)­δ_rot_(**ω̂**, **ω̂**′) = *g*(**ω̂**), and we have used the identity, *δg*(**ω̂**′)/*δg*(**ω̂**) = δ_rot_(**ω̂**, **ω̂**′). Thus, we obtain

20 $$\frac{\delta F_{I}}{\delta \mathrm{\Gamma ̃}\left(r,\mathrm{\omega ̂},t\right)}=\frac{\delta F_{I}}{\delta \mathrm{c̃}\left(r,t\right)}+\mathrm{\omega ̂}\cdot \frac{\delta F_{I}}{\delta \mathrm{P̃}\left(r,t\right)}$$

Now, we are in a position to derive the equations for surfactant concentration and polarization. Consider first the equation for the concentration *c̃*(**r**, *t*) which from eq 17, and integrating eq 16 over **ω̂**, yields

21 $$\partial_{t}c=\nabla_{r}\cdot \left[\frac{h^{2}c}{2\eta }\nabla_{r}\frac{\delta F_{I}}{\delta h}-\frac{h}{\eta }c\nabla_{r}\gamma \right]+\nabla_{r}\cdot \left[\left(D_{∥}-D_{⊥}\right)Q^{\mathrm{xy}}\cdot \nabla_{r}\frac{\delta F_{I}}{\delta \mathrm{c̃}}\right]+\nabla_{r}\cdot \left[\left(\frac{D_{∥}}{3}+\frac{2D_{⊥}}{3}\right)c\nabla_{r}\frac{\delta F_{I}}{\delta \mathrm{c̃}}\right]+\nabla_{r}\cdot \left[D_{⊥}\left(P\cdot \nabla_{r}\right)\frac{\delta F_{I}}{\delta \mathrm{P̃}}\right]$$

where the rotational term in eq 16 integrates to zero and we have assumed moments higher-than-second are small and can be neglected. This assumption may be made due to the rapid relaxation of higher angular modes under rotational diffusion and the strong anchoring due to the amphiphilic nature of the surfactants. This means that these moments decay faster than the lower ones, and thus, they may be neglected under the adiabatic approximation. Recall, *D*_⊥_ and *D*_∥_ are the perpendicular and parallel diffusion coefficients from the diffusion tensor in eq 2, respectively. We have also taken the interfacial tension γ to be independent of **ω̂** (although it can be dependent on *c* and **P**). **Q**(**r**, *t*) is a traceless symmetric tensor defined by,^38,39^

22 $$Q\left(r,t\right)=\oint d\mathrm{\omega ̂}\left(\mathrm{\omega ̂}⊗\mathrm{\omega ̂}-\frac{1}{3}I\right)\Gamma \left(r,\mathrm{\omega ̂},t\right)$$

which describes the local alignment of the particle axes without distinguishing head from tail and vanishes in the isotropic case.^39^ Thus, **Q** ^*xy*^ is the block matrix of the *x*- and *y*-components of **Q**.

Similarly, using eq 18 we may average over eq 16 to obtain the polarization thin-film equation (details are given in Appendix B)­

23 $$\partial_{t}P=\nabla_{r}\cdot \left[\frac{h^{2}}{2\eta }P⊗\nabla_{r}\frac{\delta F_{I}}{\delta h}-\frac{h}{\eta }P⊗\nabla_{r}\gamma \right]+\nabla_{r}\cdot \left[D_{⊥}P⊗\nabla_{r}\frac{\delta F_{I}}{\delta \mathrm{c̃}}\right]+D_{\mathrm{rot}}\left(Q-\frac{2}{3}\mathrm{cI}\right)\cdot \frac{\delta F_{I}}{\delta \mathrm{P̃}}$$

where again we have neglected all moments higher than the second. Interestingly, in eq 26 the rotation term does contribute to the dynamics, and is in a nonconserved form.

Equations 24 and 26 along with the equation for the film height, eq 14, comprise the set of equations that describe the dynamics of a thin liquid film with a surfactant-laden interface, where the surfactants are orientable. However, in their current state, this set of equations is not closed and hence not particularly useful. The occurrence of the tensor **Q** means we have to supplement our set of equations with a governing equation for **Q**. However, this equation will itself contain higher-order moments, and we will have an infinite hierarchy of moment equations, which means we are still not able to get closure. In the case of nematic liquid crystals many closure approximations exist, e.g., refs 40–42 and references therein. In these cases the fluid is apolar and thus the tensor **Q** is the lowest-order moment of interest, such that the moment closures are concerned with higher-order tensors. In our case, the surfactants are strongly polar, so we are concerned with a moment closure for **Q** as the lower-order moment **P** is nonzero. Therefore, we assume that the polarization is dominant and that **Q** relaxes quickly, in comparison, and thus may be adiabatically slaved to the polarization **P** (and concentration), through some function **q**, i.e., **Q** ≈ **q**(*c*,**P**). **q**(*c*, **P**) may be approximated by making the argument that the surfactants are strongly aligned perpendicular to the surface, which seems reasonable for surfactants, due to them being strongly polar. So, when the surfactants are exactly aligned perpendicular to the film surface, we may take that **q** be written in terms of the surface normal **n̂** as

24 $$q=c\left(\mathrm{n̂}⊗\mathrm{n̂}-\frac{1}{3}I\right)$$

Now, if we take that the polarization may be written in terms of the surface normal plus a small perturbation, i.e., **P** = –*c* **n̂** + δ**P**, then for δ**P** ≪ *c*, up to leading order we may write q=P⊗P/c−cI/3+O(δP/c) . Thus, under the assumption of strong polar alignment of the surfactants, we may write the leading-order approximation to the tensor **Q**, slaved to *c* and **P**, as

25 $$Q\left(r,t\right)\approx \frac{P\left(r,t\right)⊗P\left(r,t\right)}{c\left(r,t\right)}-\frac{c\left(r,t\right)}{3}I$$

## Interfacial Free-Energy Functional

In the thin-film eqs 14, 24, and 26, the interfacial free energy is yet to be defined, thus we must express this functional explicitly in terms of the height, surfactant concentration and polarization to be able to carry out the functional derivatives that follow. The free-energy functional will be made up of different contributions, arising from different aspects of the system, which in turn depend on the different observables present, namely, film height, surfactant concentration and polarization. The only contribution that depends purely on the height of the film is from the binding potential^43^ Φ­(*h*), which describes the interaction between the substrate and the film, and is related to the so-called disjoining or Derjaguin pressure going back to Derjaguin^44,45^ and Frumkin,^46^ Π = – Φ′(*h*), valid for parallel or nearly parallel interfaces (e.g., liquid–vapor, liquid–solid as is the case here); it is the extra pressure acting on a substrate due to the presence of a thin liquid film, e.g., refs 47–49 .

Let us note that one may include concentration dependence in the binding potential which will introduce an extra term to the Marangoni force.^15,16^ Then, we have the contribution due to the surfactant molecules on the surface through the local interfacial free energy^16^ *f*_*s*_(*c*, **P**), and we include a term to penalise spatial inhomogeneities in surfactant concentration which is to account for surfactant phase transitions.^2,14^ Similarly, we have the contributions from the surfactant polarization which can emerge from the energy of spontaneous polarization, elastic energy^39^ and the anchoring of the hydrophilic heads of the surfactant to the film surface. Additionally, one may include contributions from the coupling between the different observables,^50,51^ however, these shall be omitted: our aim is to consider a minimal model that captures the main ingredients of the system by focusing on the most significant effects. Thus, the interfacial free-energy functional is written as

FI[h,c,P]=∫dr[Φ(h)+ξ(fs(c,P)+κc2|∇sc|2)+ξ(a2|Pc|2+b4|Pc|4+κP2(∇sPc):(∇sPc)+WPc·n̂)] 26

Here κ_*c*_ is the interfacial rigidity constant and appears in the term responsible for penalising concentration gradients, and is also found in the free-energy associated with the Cahn–Hilliard equation.^52^ The parameters *a* and *b* arise from the spontaneous polarization term, where in the case of surfactants this originates from the orientational entropy,^19^ such that *a* = 3*k*_*B*_ *Tc*, and the quartic term is present, with *b* > 0, for stability to prevent **P** growing uncontrollably and to include saturation effects.^39^ κ_*P*_ is the Frank elastic constant^39^ which is typically of the order^53^ κ_*P*_ ∼ 10^–12^N, where the associated term penalises large gradients in the orientation of the surfactants, smoothing out the polarization field. *W* is the anchoring strength, where *W* > 0 favors the head of the surfactant molecules pointing into the surface, that is the polarization field is antiparallel to the surface normal, which accounts for the hydrophilic head and hydrophobic tail of the surfactant,^54^ and *W* is typically of the order^55^ *W* ∼ 10^–6^J/m^2^. The local interfacial free energy *f*_*s*_(*c*, **P**) is given in terms of different contributions as^16^

27 $$f_{s}\left(c,P\right)=\gamma_{0}+\frac{k_{B}T}{l^{2}}c\left(\ln c-1\right)+f_{s}^{\mathrm{exc}}\left(c,P\right)$$

where γ_0_ is the surface tension in the absence of surfactants, the second term in eq 30 is the ideal-gas contribution, and *f*_*s*_^exc^(*c*, **P**) is the excess-overideal free energy which accounts for interactions between surfactant molecules (see eqs 5 and 6). Here we assume that the contribution of the surfactant orientation to the local excess free energy is entirely through the polarization field.

From eq 29 we may compute the functional derivatives in eqs 14, 24, and 26 to fully close this set of equations. Recalling, we must replace *c* and **P** by *c̃*/ξ and **P̃**/ξ, respectively, in eq 29 to perform the variations. After doing so, and by taking the leading-order terms in *ε*, in accordance with the long-wave approximation, *ε* ∼ | ***∇***_**r**_ *h*| ≪ 1 such that we take ξ ≈ 1, we find the variational derivative with respect to the liquid film height as^16^

$$\frac{\delta F_{I}}{\delta h\left(r,t\right)}=\partial_{h}\Phi \left(h\right)-\nabla_{r}\cdot \left[(f_{s}\left(c,P\right)-c\partial_{c}\;f_{s}\left(c,P\right)-P\cdot \nabla_{P}\;f_{s}\left(c,P\right)-\frac{\kappa_{c}}{2}{\left|\nabla_{r}c\right|}^{2}+\kappa_{c}c\nabla_{r}^{2}c)\nabla_{r}h\right]-\nabla_{r}\cdot \left[(\frac{a}{2}\frac{{\left|P\right|}^{2}}{c^{2}}+\frac{b}{4}\frac{{\left|P\right|}^{4}}{c^{4}}+\frac{\kappa_{P}}{2}(\nabla_{r}\frac{P}{c})\;:\left(\nabla_{r}\frac{P}{c}\right)+W\frac{P}{c}\cdot \mathrm{n̂})\nabla_{r}h\right]$$ 28

where under the long-wave approximation we have replaced the surface gradient with the *d* = 2 Cartesian gradient, ***∇***_**r**_. The variations with respect to *c̃* and **P̃** are

29 $$\frac{\delta F_{I}}{\delta \mathrm{c̃}\left(r,t\right)}=\partial_{c}\;f_{s}\left(c,P\right)-\kappa_{c}\nabla_{r}^{2}c-\frac{1}{c}\left(a\frac{{\left|P\right|}^{2}}{c^{2}}+b\frac{{\left|P\right|}^{4}}{c^{4}}\right)+\kappa_{P}\frac{P}{c^{2}}\cdot \nabla_{r}^{2}\frac{P}{c}-W\frac{P}{c^{2}}\cdot \mathrm{n̂}$$

$$\frac{\delta F_{I}}{\delta \mathrm{P̃}\left(r,t\right)}=\nabla_{P}\;f_{s}\left(c,P\right)+P\left(\frac{a}{c^{2}}+b\frac{|P{|}^{2}}{c^{4}}\right)-\kappa_{P}\frac{1}{c}\nabla_{r}^{2}\frac{P}{c}+W\frac{1}{c}\mathrm{n̂}$$ 30

Let us now define the generalized interfacial tension, γ, as

31 γ=fs(c,P)−c∂cfs(c,P)−P·∇Pfs(c,P)−κc2|∇rc|2+κcc∇r2c+a2|P|2c2+b4|P|4c4+κP2(∇rPc):(∇rPc)+WPc·n̂

where the terms involving **P** are in addition to the usual form of γ when surfactant orientability is not considered.^2^ From eq 31 we then have that

32 $$\frac{\delta F_{I}}{\delta h\left(r,t\right)}=\partial_{h}\Phi \left(h\right)-\nabla_{r}\cdot \left(\gamma \nabla_{r}h\right)$$

This definition of the surface tension, eq 34, can be understood by proving the equivalence between the (generalized) surface tension and the (generalized) local surface grand-potential density,^15^ i.e., γ = ω_*s*_, where the surface grand potential is given as

$$\Omega_{s}\left[c,P\right]=\int dr\xi \omega_{s}=\int dr\xi \left(g_{s}\left(c,P\right)-c\mu_{s}-P\cdot O\right)$$ 33

where μ_*s*_ is the surface-chemical potential and **O** is the orientation field potential, i.e., the conjugate field to the polarization, **P**. *g*_*s*_(*c*, **P**) is the Helmholtz free energy due to the presence of the orientable surfactants, such that, as compared to eq 29 the free-energy functional is written as

$$F_{I}\left[h,c,P\right]=\int dr\left[\Phi \left(h\right)+\xi g_{s}\left(c,P\right)\right]$$ 34

The surface chemical potential and orientation potential field are related to their conjugate variables via the variational derivatives

35 $$\mu_{s}=\frac{\delta F_{I}}{\delta \mathrm{c̃}},\mathrm{and},O=\frac{\delta F_{I}}{\delta \mathrm{P̃}}$$

which from inserting eqs 32 and 33, along with the local surface free energy, *g*_*s*_, defined in eqs 37 and 29, into eq 36, and from the surface tension in eq 34, one obtains

$$\Omega_{s}\left[c,P\right]=\int dr\xi \gamma \left(c,P\right)$$ 36

which gives the equivalence between the surface tension and local grand potential, γ = ω_*s*_, as required.

The result of this equivalence means one can connect with thermodynamics and derive a Gibbs–Duhem like equation (at constant temperature) for the gradient of the surface tension, i.e., from eqs 32, 33, and 34 it can be shown that

$$\nabla_{r}\gamma =-c\nabla_{r}\frac{\delta F_{I}}{\delta \mathrm{c̃}\left(r,t\right)}-\left(P\cdot \nabla_{r}\right)\frac{\delta F_{I}}{\delta \mathrm{P̃}\left(r,t\right)}$$ 37

Now, from eq 40 we are able to write the Marangoni flow terms, (*h*/η) *c* ***∇***_**r**_γ and (*h*/η) **P** ⊗ ***∇***_**r**_γ, in eqs 24 and 26, respectively in terms of the free-energy functional, meaning the set of thin-film equations 14, 24, and 26, may be represented fully in a gradient dynamics form as

38a $$\partial_{t}h=\nabla_{r}\cdot \left[\frac{h^{3}}{3\eta }\nabla_{r}\frac{\delta F_{I}}{\delta h}+\frac{h^{2}}{2\eta }\left(c\nabla_{r}\frac{\delta F_{I}}{\delta \mathrm{c̃}}+\left(P\cdot \nabla_{r}\right)\frac{\delta F_{I}}{\delta \mathrm{P̃}}\right)\right]$$

38b $$\partial_{t}c=\nabla_{r}\cdot \left[\frac{h^{2}c}{2\eta }\nabla_{r}\frac{\delta F_{I}}{\delta h}+\frac{h}{\eta }c\;\left(c\nabla_{r}\frac{\delta F_{I}}{\delta \mathrm{c̃}}+\left(P\cdot \nabla_{r}\right)\frac{\delta F_{I}}{\delta \mathrm{P̃}}\right)\right]+\nabla_{r}\cdot \left[\left(D_{∥}-D_{⊥}\right)Q^{\mathrm{xy}}\cdot \nabla_{r}\frac{\delta F_{I}}{\delta \mathrm{c̃}}\right]+\nabla_{r}\cdot \left[\left(\frac{D_{∥}}{3}+\frac{2D_{⊥}}{3}\right)\;c\nabla_{r}\frac{\delta F_{I}}{\delta \mathrm{c̃}}\right]+\nabla_{r}\cdot \left[D_{⊥}\left(P\cdot \nabla_{r}\right)\frac{\delta F_{I}}{\delta \mathrm{P̃}}\right]$$

38c $$\partial_{t}P=\nabla_{r}\cdot \left[\frac{h^{2}}{2\eta }P⊗\nabla_{r}\frac{\delta F_{I}}{\delta h}+\frac{h}{\eta }P⊗\left(c\nabla_{r}\frac{\delta F_{I}}{\delta \mathrm{c̃}}+\left(P\cdot \nabla_{r}\right)\frac{\delta F_{I}}{\delta \mathrm{P̃}}\right)\right]+\nabla_{r}\cdot \left[D_{⊥}P⊗\nabla_{r}\frac{\delta F_{I}}{\delta \mathrm{c̃}}\right]+D_{\mathrm{rot}}\;\left(Q-\frac{2}{3}\mathrm{cI}\right)\cdot \frac{\delta F_{I}}{\delta \mathrm{P̃}}$$

For the conserved quantities *h* and *c* their governing equations are in a conservative form, as is the case in nonpolar surfactant molecules, or molecules with low polarizability, whereas for the polarization **P** the rotational diffusion associated with the surfactants induces a nonconserved contribution. The Marangoni flux in eq 41 is given in the usual form,^15^ – (*h* ^2^/2η) ***∇***_**r**_γ (from eq 40), however in this case, the surface tension is of a more generalized form, eq 34, such that it is dependent not only on surfactant concentration but surfactant polarization as well, i.e., γ­(*c*, **P**).

These evolution equations are consistent with the film-height-surfactant equations in the absence of the additional effects considered here. Indeed, if we take the surfactants as symmetric point-like particles, the polarization vanishes, *D*_∥_ = *D*_⊥_ = *D* and $D_{\mathrm{rot}}=0$ , such that eq 38 reduces to the previously found set of coupled equations for the film height and concentration,^15^ which are given as,

39a $$\partial_{t}h=\nabla_{r}\cdot \left[\frac{h^{3}}{3\eta }\nabla_{r}\frac{\delta F_{I}}{\delta h}+\frac{h^{2}c}{2\eta }\nabla_{r}\frac{\delta F_{I}}{\delta \mathrm{c̃}}\right]$$

39b $$\partial_{t}c=\nabla_{r}\cdot \left[\frac{h^{2}c}{2\eta }\nabla_{r}\frac{\delta F_{I}}{\delta h}+\frac{hc^{2}}{\eta }\nabla_{r}\frac{\delta F_{I}}{\delta \mathrm{c̃}}\right]+\nabla_{r}\cdot \left[\mathrm{Dc}\nabla_{r}\frac{\delta F_{I}}{\delta \mathrm{c̃}}\right]$$

## Summary and Conclusions

We have considered the case of a surfactant-laden thin liquid film, with the surfactant molecules being treated as anisotropic, such that they have a head–tail polar structure and diffuse across the film surface as well as rotationally. We have presented a microscopic derivation of the interface transport equation for such molecules, using elements from the statistical mechanics of classical fluids, namely DFT, and its dynamic extension, DDFT.

First, we introduced the general DDFT for uniaxial orientable colloids and generalized this to the case in which this dynamics is occurring on the surface of a thin film. Invoking the long-wave approximation we are able to find the equation governing the joint surfactant concentration and orientation density, which is coupled to the hydrodynamic equation for the film height that includes the gradient of interfacial tension. We take that the only contributions to the free-energy functional associated with the surfactant dynamics are through the surfactant concentration and polarization field, which are appropriately defined as the zeroth- and first-moment of the joint distribution with respect to the orientation. We then take the respective moments of the governing equation to find thin-film equations for the surfactant concentration and polarization field. To fully close this set of equations, we express the free energy-functional in its explicit form in terms of the film height, surfactant concentration and polarization, where there are contributions from the disjoining pressure, the free energy of the surfactants, as well as terms related to the polarization of the surfactants. After computing the variations of the free-energy functional with respect to the fields of interest, and defining a generalized surface tension, we finally arrive at the set of equations for a thin film laden with orientable polar uniaxial surfactants. These evolution equations, are of gradient dynamics form, as they should be, and they reduce to the usual equations for nonpolar surfactants.

There are a number of interesting questions related to the analysis presented here, and further avenues for which the results may be explored. First, it would be interesting to investigate how to close the set of thin-film equations 38, via the approximation of the **Q** alignment tensor through the polarization **P**, where existing techniques from liquid-crystal theory may be useful.^38^ Alternatively, one could truncate the moment hierarchy at a higher order, i.e., derive an additional equation for **Q**, and appropriately devise closure approximations for the higher moments. It would also be interesting to allow the surfactants to be soluble such that an extra observable, the dissolved surfactant density, would need to be included in the formulation. In this case, the orientation of the surfactants would have a significant impact on surface adsorption; in particular, the adsorption flux^16^ can be taken to be dependent on the polarization, leading to a more realistic model for thin films with soluble surfactants.

The utility of the gradient form of the thin-film equations allows one to simply extend the free-energy functional to more accurate approximations, namely, weighted-density approximations, fundamental measure theory, statistical associating fluid theory and molecular DFT, e.g., refs 22, 48 and 56–60 , as well as including higher-order polarization induced contributions to the free-energy functional.^39,50,51^ Furthermore, with recent advances in the application of statistical-learning techniques to DFT^61–64^ and beyond,^65,66^ it would be interesting to apply these to gain a greater understanding of the effect of surfactant orientation on the dynamics. In particular, it would be interesting to use molecular dynamics of orientable surfactants to try and glean the structure of the free-energy functional via functional mappings which would go beyond the adiabatic approximation.^28,67^

On the hydrodynamics front, the majority of thin-film studies are typically based on macroscopic models, as done here. There are some notable exceptions, e.g., the recent work in ref 68 which offered a microscopic derivation of the thin-film equation using the Mori-Zwanzig projection-operator formalism. Of particular interest here, would be a fully microscopic derivation, where in addition to the surfactant interfacial transport equation, we also have a microscopic derivation of the hydrodynamic equation for the film height which involves the gradient of interfacial tension. Extending these equations to allow for stochasticity-noise would enable scrutiny of the effects of thermal fluctuations, e.g., ref 69. Further, one may include nonconserved dynamics such as evaporation/condensation^16^ or multiple species of surfactants,^17^ which could be taken to be dependent on the surfactant polarization; or indeed additional levels of complexity such as reactive surfactants,^70^ where the coupling of thin-film hydrodynamics and surfactant chemistry can either stabilize instabilities occurring in the pure chemical subsystem, or indeed it can induce instabilities in regions where the pure hydrodynamic and chemical subsystems are both stable. We shall examine these and related problems in future studies.

## Appendix A. Derivation of Eq 7

We show how we derive the surface DDFT eq 7 from eq 1. We start by considering the continuity equation for the conserved surfactant concentration diffusing on the free surface, *S*(*t*),

ddt∫S(t)dS∫Ωdω̂Γ(r,ω̂,t)+∫∂S(t)dls∫Ωdω̂J(r,ω̂,t)·n̂s+∫S(t)dS∫∂Ωdlω̂J(r,ω̂,t)·n̂Ω=0
40

where *d*/*dt* = ∂/∂*t* + **v**_*s*_· ***∇***_*s*_ is the material derivative (see comments below eq 8 and ref 70 about the subtleties of this material derivative). **J**(**r**, **ω̂**, *t*) and J(r,ω̂,t) are the fluxes associated with the translational diffusion and rotational diffusion, respectively. Ω is an enclosed region of orientation space, i.e., a subset of the unit sphere, and $dl_{s}$ and $dl_{\mathrm{\omega ̂}}$ are the line elements on the surface and unit sphere, respectively. **n̂**_*s*_ and **n̂**_Ω_ are the unit normal vectors to *S* and Ω, respectively. Using the divergence theorem for surfaces and spheres, we have

41
ddt∫S(t)dS∫Ωdω̂Γ(r,ω̂,t)+∫S(t)dS∫Ωdω̂[∇s·J(r,ω̂,t)+∇ω̂·J(r,ω̂,t)]=0

Now, we may bring the material derivative inside the integral, but as the surface is evolving, we must use the product rule along with the identity,^71^

42
$$\frac{d}{\mathrm{dt}}\mathrm{dS}=\mathrm{dS}\nabla_{s}\cdot v$$

to obtain,

43
∫S(t)dS∫Ωdω̂[∂∂tΓ+∇s·(vΓ)]+∫S(t)dS∫Ωdω̂[∇s·J(r,ω̂,t)+∇ω̂·J(r,ω̂,t)]=0

By writing eq 1 in the form of a continuity equation,

44
$$\partial_{t}\rho \left(r,\mathrm{\omega ̂},t\right)=-\nabla_{r}\cdot J\left(r,\mathrm{\omega ̂},t\right)-\nabla_{\mathrm{\omega ̂}}\cdot J\left(r,\mathrm{\omega ̂},t\right)$$

we are able to glean the expressions for the fluxes as,

45a
$$J\left(r,\mathrm{\omega ̂},t\right)=-D\left(\mathrm{\omega ̂}\right)\cdot \rho \left(r,\mathrm{\omega ̂},t\right)\nabla_{r}\frac{\delta F}{\delta \rho }$$

45b
$$J\left(r,\mathrm{\omega ̂},t\right)=D_{\mathrm{rot}}\mathrm{\omega ̂}\times \left(\rho \left(r,\mathrm{\omega ̂},t\right)\mathrm{\omega ̂}\times \nabla_{\mathrm{\omega ̂}}\frac{\delta F}{\delta \rho }\right)$$

where we have used

$$\left(\mathrm{\omega ̂}\times \nabla_{\mathrm{\omega ̂}}\right)\cdot \left(\rho \mathrm{\omega ̂}\times \nabla_{\mathrm{\omega ̂}}\delta F/\delta \rho \right)=-\nabla_{\mathrm{\omega ̂}}\cdot \left[\mathrm{\omega ̂}\times \left(\rho \mathrm{\omega ̂}\times \nabla_{\mathrm{\omega ̂}}\delta F/\delta \rho \right)\right]$$

Now, by constraining these fluxes to the surface *z* = *h*(**r**, *t*), we have

J(r,ω̂,t)→J(r,ω̂,t)

J(r,ω̂,t)→J(r,ω̂,t)

and $\nabla_{r}\to \nabla_{s}$ , then writing in terms of the concentration Γ(**r**, **ω̂**, *t*) and inserting into eq 49, we find

$$\begin{array}{l} \frac{\partial }{\partial t}\Gamma +\nabla_{s}\cdot \left(v\Gamma \right)=\nabla_{s}\cdot \left[D_{s}\left(\mathrm{\omega ̂}\right)\cdot \Gamma \nabla_{s}\frac{\delta F}{\delta \Gamma }\right]-D_{\mathrm{rot}}\nabla_{\mathrm{\omega ̂}}\cdot \left[\mathrm{\omega ̂}\times \left(\Gamma \mathrm{\omega ̂}\times \nabla_{\mathrm{\omega ̂}}\frac{\delta F}{\delta \Gamma }\right)\right]\\ =\nabla_{s}\cdot \left[D_{s}\left(\mathrm{\omega ̂}\right)\cdot \Gamma \nabla_{s}\frac{\delta F}{\delta \Gamma }\right]+D_{\mathrm{rot}}\mathrm{\omega ̂}\times \nabla_{\mathrm{\omega ̂}}\cdot \left[\Gamma \mathrm{\omega ̂}\times \nabla_{\mathrm{\omega ̂}}\frac{\delta F}{\delta \Gamma }\right] \end{array}$$
46

where we have neglected the integrals as Γ is conserved and the surface and orientation is arbitrary. Finally, by writing **v** in terms of its tangential, **v**_*s*_ = (**I** – **n̂** ⊗ **n̂**)·**v**, and normal, **v**_*n*_ = (**v**·**n̂**)**n̂**, components, we obtain eq 7.

## Appendix B. Calculation of Rotational Term in eq 26

Here, we illustrate how taking the average over the final term on the right-hand side (RHS) of eq 16 with respect to **ω̂**, via eq 18, leads to the final term on the RHS of eq 26. Inserting eq 23 into eq 16 leads to the expression for the final term as,

47
$$D_{\mathrm{rot}}\mathrm{\omega ̂}\times \nabla_{\mathrm{\omega ̂}}\cdot \left[\Gamma \mathrm{\omega ̂}\times \nabla_{\mathrm{\omega ̂}}\frac{\delta F_{I}}{\delta \mathrm{\Gamma ̃}}\right]=D_{\mathrm{rot}}\mathrm{\omega ̂}\times \nabla_{\mathrm{\omega ̂}}\cdot \left[\Gamma \mathrm{\omega ̂}\times \frac{\delta F_{I}}{\delta \mathrm{P̃}}\right]$$

Averaging over eq 54 with respect to **ω̂** gives,

48
$$\begin{array}{l} D_{\mathrm{rot}}\oint d\mathrm{\omega ̂}\mathrm{\omega ̂}\left(\mathrm{\omega ̂}\times \nabla_{\mathrm{\omega ̂}}\right)\cdot \left[\Gamma \mathrm{\omega ̂}\times \frac{\delta F_{I}}{\delta \mathrm{P̃}}\right]=D_{\mathrm{rot}}\oint d\mathrm{\omega ̂}\mathrm{\omega ̂}\left[\mathrm{\omega ̂}\cdot \left(\nabla_{\mathrm{\omega ̂}}\times \left(\Gamma \mathrm{\omega ̂}\times \frac{\delta F_{I}}{\delta \mathrm{P̃}}\right)\right)\right]\\ =-D_{\mathrm{rot}}\oint d\mathrm{\omega ̂}\mathrm{\omega ̂}\left[\nabla_{\mathrm{\omega ̂}}\cdot \left(\mathrm{\omega ̂}\times \left(\Gamma \mathrm{\omega ̂}\times \frac{\delta F_{I}}{\delta \mathrm{P̃}}\right)\right)\right] \end{array}$$

since ***∇***_**ω̂**_ × **ω̂** = **0**. Now, by integrating by parts we obtain,

49
$$\begin{array}{l} -D_{\mathrm{rot}}\oint d\mathrm{\omega ̂}\mathrm{\omega ̂}\left[\nabla_{\mathrm{\omega ̂}}\cdot \left(\mathrm{\omega ̂}\times \left(\Gamma \mathrm{\omega ̂}\times \frac{\delta F_{I}}{\delta \mathrm{P̃}}\right)\right)\right]=D_{\mathrm{rot}}\oint d\mathrm{\omega ̂}\left(\nabla_{\mathrm{\omega ̂}}\mathrm{\omega ̂}\right)\cdot \left(\mathrm{\omega ̂}\times \left(\Gamma \mathrm{\omega ̂}\times \frac{\delta F_{I}}{\delta \mathrm{P̃}}\right)\right)\\ =D_{\mathrm{rot}}\oint d\mathrm{\omega ̂}\left(I-\mathrm{\omega ̂}⊗\mathrm{\omega ̂}\right)\cdot \left[\mathrm{\omega ̂}\left(\Gamma \mathrm{\omega ̂}\cdot \frac{\delta F_{I}}{\delta \mathrm{P̃}}\right)-\Gamma \frac{\delta F_{I}}{\delta \mathrm{P̃}}\right] \end{array}$$

where we have used that ***∇***_**ω̂**_ **ω̂** = **I** – **ω̂** ⊗ **ω̂** as ***∇***_**ω̂**_ is the gradient on the unit sphere. We have also made use of the triple-cross-product expansion along with the fact that **ω̂** is a unit vector.

It can be shown that the first term vanishes, i.e.,

50
$$\left(I-\mathrm{\omega ̂}⊗\mathrm{\omega ̂}\right)\cdot \mathrm{\omega ̂}\left(\Gamma \mathrm{\omega ̂}\cdot \frac{\delta F_{I}}{\delta \mathrm{P̃}}\right)=\mathrm{\omega ̂}\left(\Gamma \mathrm{\omega ̂}\cdot \frac{\delta F_{I}}{\delta \mathrm{P̃}}\right)-\mathrm{\omega ̂}\left(\mathrm{\omega ̂}\cdot \mathrm{\omega ̂}\left(\Gamma \mathrm{\omega ̂}\cdot \frac{\delta F_{I}}{\delta \mathrm{P̃}}\right)\right)=0$$

and thus we find

51
$$\begin{array}{l} D_{\mathrm{rot}}\oint d\mathrm{\omega ̂}\left(I-\mathrm{\omega ̂}⊗\mathrm{\omega ̂}\right)\cdot \left[\mathrm{\omega ̂}\left(\Gamma \mathrm{\omega ̂}\cdot \frac{\delta F_{I}}{\delta \mathrm{P̃}}\right)-\Gamma \frac{\delta F_{I}}{\delta \mathrm{P̃}}\right]=-D_{\mathrm{rot}}\oint d\mathrm{\omega ̂}\left(I-\mathrm{\omega ̂}⊗\mathrm{\omega ̂}\right)\cdot \Gamma \frac{\delta F_{I}}{\delta \mathrm{P̃}}\\ =D_{\mathrm{rot}}\left(Q-\frac{2}{3}c\right)\frac{\delta F_{I}}{\delta \mathrm{P̃}} \end{array}$$

where we have utilized eq 25, which gives exactly the final term on the RHS of eq 26.
